# Bedside Carotid Sinus Massage for Syncope Evaluation With Bifascicular Block and First-Degree Atrioventricular Block

**DOI:** 10.7759/cureus.33925

**Published:** 2023-01-18

**Authors:** Mashood B Badshah, Muhammad Hamza Saad Shaukat, Azar Birlas, Scott Pham

**Affiliations:** 1 Internal Medicine, University of South Dakota Sanford School of Medicine, Sioux Falls, USA; 2 Cardiology, University of South Dakota Sanford School of Medicine, Sioux Falls, USA; 3 Internal Medicine, Rehman Medical College, Peshawar, PAK

**Keywords:** permanent pacemaker, bifascicular block, atrioventricular block, cardiac syncope, carotid sinus massage

## Abstract

Symptomatic bifascicular block (BFB) with a reversible high-grade atrioventricular block (AVB) is an overlooked cause of syncope with differing diagnostic and therapeutic approaches. We present a case of a 79-year-old gentleman with multiple episodes of cardiac syncope. Initial electrocardiogram revealed a left bundle branch block and first-degree AVB worsened by bedside carotid sinus massage (CSM) obviating the need for electrophysiologic (EP) studies or continuous electrocardiographic monitoring for further evaluation. This case highlights the importance of CSM as a useful clinical tool in addition to EP studies and internal loop recorder (ILR) placement for assessment and appropriateness of permanent pacemaker (PPM) implantation. It also sheds light on the differing management protocols between EP studies and ILR evaluation versus empiric PPM implantation for patients with cardiac syncope secondary to BFB and AVB.

## Introduction

Carotid sinus massage (CSM) is a clinical maneuver frequently used for the evaluation of carotid sinus hypersensitivity due to cardioinhibitory or vasodepressor response. However, it is also a valuable clinical tool for syncope evaluation in patients with a bifascicular block (BFB) and first-degree atrioventricular block (AVB). Here, we present a case of a 79-year-old male admitted with multiple episodes of syncope, who had a BFB and first-degree AVB on electrocardiogram (ECG). He developed symptomatic high-degree AVB with bedside CSM obviating the need for further workup and was subsequently treated with a permanent pacemaker (PPM) with outpatient follow-up.

## Case presentation

**Figure 1:**
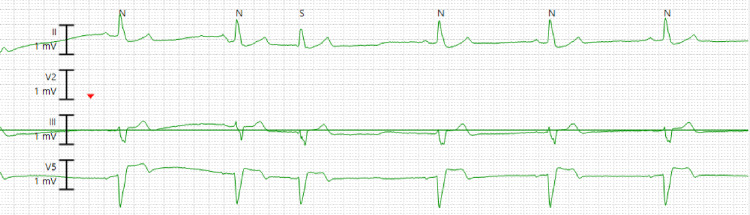
Telemetry strip showing sinus bradycardia with a heart rate of 42 beats per minute.

The patient was a pleasant 79-year-old male who was admitted to our facility with multiple episodes of syncope. The patient had a past medical history of hypertension, hyperlipidemia, Crohn's colitis on chronic vedolizumab therapy, and impaired fasting glucose. He denied any previous history of coronary artery disease, arrhythmias, ischemic/hemorrhagic stroke, or carotid artery disease. In the last month, he had endured at least three episodes of non-prodromal syncope. The last episode of syncope led to a traumatic fall and right parietal laceration, prompting admission to the hospital. After admission, his initial vital signs showed blood pressure of 136/72 mmHg, a pulse of 68 beats per minute (bpm), a respiratory rate of 18 per minute, and a temperature of 98.5°F. Physical examination revealed a right parietal scalp laceration; however, the cardiovascular examination was unremarkable. Subsequent syncope workup while admitted to the hospital, including orthostatic vitals, transthoracic echocardiography (TTE), serial troponins, and carotid Doppler, was negative for any abnormalities. However, on telemetry, he was found to have asymptomatic bradycardia at rest with his heart rate intermittently going down to as low as 42 bpm (Figure 1).

**Figure 2:**
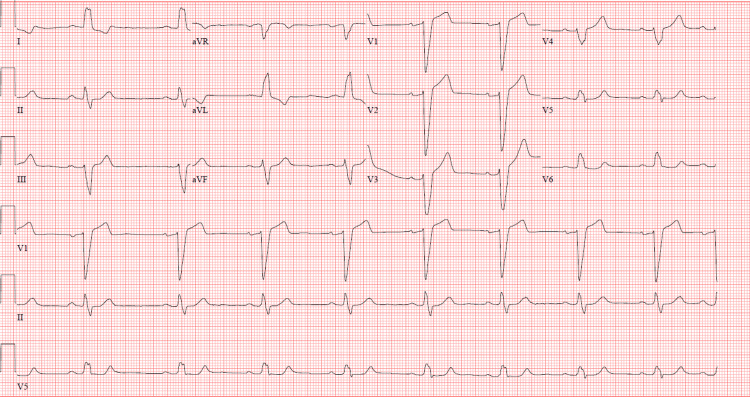
Initial ECG showing sinus bradycardia with a heart rate of 51 beats per minute, left bundle branch block with a QRS interval of 159 milliseconds (ms), and first-degree atrioventricular block with a PR interval of 208 ms.

Initial ECG showed sinus bradycardia with a heart rate of 51, a left bundle branch block (LBBB), and a first-degree atrioventricular (AV) block with a PR interval of 208 ms (Figure 2).

**Figure 3:**
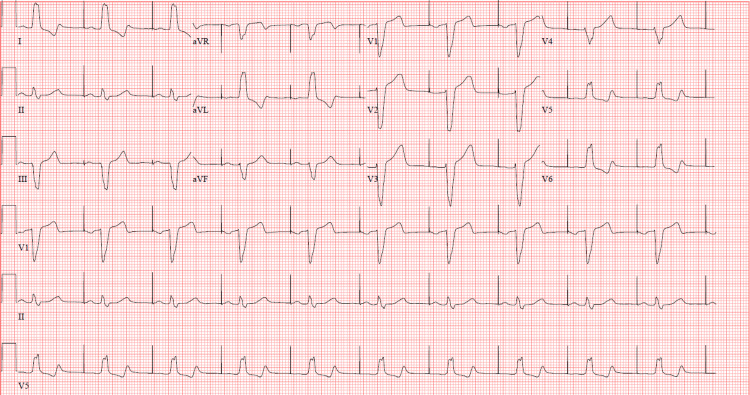
ECG after permanent pacemaker implantation showing atrial paced rhythm with underlying left bundle branch block.

Further evaluation with bedside CSM with pacer pads and emergency crash cart at bedside induced lightheadedness attributable to symptomatic bradycardia with the heart rate going to a nadir of 26 bpm on telemetry. The symptoms were relieved promptly with the termination of the CSM. Ischemic evaluation for cardiac syncope with pharmacologic nuclear stress test was unremarkable. Because of first-degree AV blockade, worsened by bedside CSM and LBBB, the patient subsequently received dual-chamber PPM with no recurrence of presyncope/syncope after the procedure. ECG after PPM placement showed an atrial paced rhythm with the heart rate at 67 bpm (Figure 3) and the patient was discharged with regular outpatient follow-up.

## Discussion

CSM is one of the most useful physical examination procedures for the evaluation of syncope. The mechanism involves stretching carotid sinus baroreceptors leading to cardioinhibitory, vasodepressor, or mixed response [1]. Cardioinhibitory response affects both the sinoatrial and AV nodes, which, if exaggerated, can lead to sinus node arrest or AVB, respectively. This exaggerated response is called carotid sinus syndrome, which is diagnosed if the patient has symptomatic or asymptomatic asystolic pause for three or six seconds, respectively [2]. CSM also has a myriad of other diagnostic and therapeutic applications including the diagnosis and treatment of supraventricular tachyarrhythmias, including atrioventricular nodal re-entrant tachycardia (AVNRT), localizing the site of type I second-degree AVB, and normalizing a bundle branch block by increasing vagal tone [1,3]. Although CSM is frequently used in reflex syncope assessment, its effectiveness in cardiac syncope diagnosis requires further data [4]. CSM is well tolerated in the elderly population, though it should be avoided in patients with carotid artery disease, transient ischemic attack, or ischemic stroke in the last three months due to the risk of stroke propagation [1,5]. Due to the risk of initiating bradyarrhythmias, including sinus nodal arrest and high-grade AV nodal block [6], the CSM should only be performed under telemetry with pacer pads and an emergency crash cart at the bedside, as in our case.

Unlike sinus nodal dysfunction and high-grade AVB, BFBs are often overlooked as a cause of underlying arrhythmia leading to syncope. Our case, while neither rare nor unusual, offers the opportunity to reflect on the clinical utility of bedside CSM and discuss the management approaches in patients with cardiogenic syncope secondary to BFB and reversible high-grade AVB. Although BFB, including LBBB and right bundle branch block (RBBB) with a left anterior or posterior fascicular block (LAFB/LPFP), is usually asymptomatic, the presence of first-degree AVB heralds the risk of complete AVB, with 13% complete AVB at a 5.4-year follow-up in one study [7]. The complete AVB risk in BFB patients warrants diagnostic evaluation; however, electrophysiologic (EP) evaluation yield is modest at 41% positivity [8] due to which implantable loop recorder (ILR) is indicated in undiagnosed cases [9]. Ischemic evaluation should also be considered part of the syncope workup due to the relationship between coronary artery disease, arrhythmia, and recurrent syncope [10].

Although intermittent complete AVB is the most common cause of syncope in patients with BFB, other etiologies may also contribute. In a study of 55 patients with syncope and bundle branch block, despite cardiac syncope being the most common cause, neurally mediated syncope was found in 40% of cases, and in 15%, the cause remained elusive [11]. A larger cohort of 323 patients with BFB and syncope while showing bradyarrhythmias, including AVB as the major cause, also revealed non-cardiogenic etiologies in a significant minority of patients, while 56 cases remain undiagnosed [12]. Empiric PPM implantation in patients with BFB and syncope is associated with an increased risk of syncope recurrence, 27% in one instance after a mean follow-up of 31 ± 21 months [13], prompting the European Society of Cardiology (ESC) to recommend initial EP and ILR evaluation before PPM placement [2]. However, ESC recommends empiric PPM placement in the elderly population due to the increased risk of syncope leading to trauma [2]. Following ESC guidelines, PPM placement in documented BFB and first-degree AVB leads to a significant reduction in symptomatic syncope [14].

Although the American College of Cardiology/American Heart Association/Heart Rhythm Society (ACC/AHA/HRS) guidelines also advise ECG monitoring and EP studies in syncope patients with bundle branch disease [15], the presence of BFB in symptomatic patients herald an increased risk of high-degree AVB justifying empiric PPM implantation [16]. Studies comparing empiric PPM versus EP evaluation in BFB patients with syncope revealed decreased syncope recurrence and AVB in patients who were treated with empiric PPM; however, overall mortality was similar [17]. A recent trial comparing empiric PPM placement versus ILR evaluation in patients with BFB and syncope revealed decreased primary composite outcome of bradyarrhythmia, device complications, or cardiovascular death in the PPM group indicating superiority of empiric PPM implantation strategy (35% in PPM vs. 76% in ILR group; p < 0.0001) [18]. The syncope recurrence remained similar between empiric PPM vs. ILR groups since the main driver was thought to be due to the vasodepressor response [18]. Cardiac PPM implantation was appropriate in our case where the cause of syncope with BFB was confirmed to be intermittent high-grade AVB on telemetry while performing bedside CSM obviating the need for further EP studies or ILR placement.

After PPM implantation, there remains a risk for syncope recurrence in bradyarrhythmic syncope patients including patients with syncope and BFB, with a recent study indicating 15.6% recurrence at 50 months follow-up [19]. The risk of syncope recurrence is highest for patients who underwent PPM placement for vasovagal syncope (26.4%) followed by patients with unexplained syncope and chronic BFB (21.5%) [19]. Overall, the most common cause of recurrence was reflex syncope (27.7%), followed by orthostatic hypotension (26.3%). However, in patients with PPM placement for syncope and BFB, 29.4% of recurrences remained unexplained while 26.5% of recurrences were due to reflex syncope [19]. The risk of recurrence mandates a discussion of risks and benefits, before PPM implantation for patients with syncope, first-degree AVB, and BFB.

## Conclusions

In essence, the CSM is an inexpensive bedside maneuver for intermittent high-risk AVB diagnosis, which can circumvent the need for further testing including EP studies and ILR placement in patients with BFB and syncope. Further studies are needed to quantify the role of bedside CSM under telemetry in assessing the risk of AVB in BFB patients. Although current guidelines recommend EP studies and ILR evaluation before PPM placement, further studies are also required to evaluate the utility of empiric PPM placement in symptomatic AVB with BFB in patients.
